# Characteristics of retinal image associated with premature ovarian insufficiency: a case- control study

**DOI:** 10.1186/s13048-023-01231-0

**Published:** 2023-07-24

**Authors:** Jiaman Wu, Liya Tan, Yan Ning, Weiqu Yuan, Zuowei Lee, Fei Ma, Erfeng Wang, Yuanyuan Zhuo

**Affiliations:** 1 Affiliated Shenzhen Maternity & Child Healthcare Hospital, Southern Medical University, Shenzhen, 518028 China; 2 Guangzhou University of Chinese Medicine, Guangzhou, China; 3 Shenzhen Traditional Chinese Medicine Hospital, Shenzhen, 518033 China; 4 Division of Biostatistics, Jockey Club School of Public Health and Primary Care, The Chinese University of Hong Kong, Hong Kong, China; 5 Centre for Clinical Trials and Biostatistics Lab, CUHK Shenzhen Research Institute, Shenzhen, China

**Keywords:** Retinal characteristics, Premature ovarian insufficiency, Retinal image analysis, Biostatistics method, Early clinical diagnosis

## Abstract

**Purpose:**

To establish an early clinical diagnosis model based on the retinal vascular features associated with POI, supplying a non-invasive way for accurately and early predicted the risk of POI.

**Methods:**

A total of 78 women with spontaneous POI and 48 healthy women were recruited from the Affiliated Shenzhen Maternity & Child Healthcare Hospital in the study. Retinal characteristics were analyzed using an automated retinal image analysis system. Binary logistic regression was used to identify POI cases and develop predictive models.

**Results:**

Compared to the normal group, the POI group had larger central retinal artery equivalent (CRAE) (*P* = 0.006), central retinal vein equivalent (CRVE) (*P* = 0.001), index of venules asymmetry (Vasym) (*P* = 0.000); larger bifurcation angles of arterioles (Aangle) (*P* = 0.001), bifurcation coefficient of venule (BCV) (*P* = 0.001) and more obvious arteriovenous nipping (Nipping) (*P* = 0.005), but lower arteriole-to-venule ratio (AVR) (*P* = 0.012). In the POI group, the odds ratio (OR) of Vasym was 6.72e-32 (95% C.I. 4.62e-49–9.79e-15, *P* = 0.000), the OR of BCV was 5.66e-20 (95% C.I. 1.93e-34–.0000, *P* = 5.66e-20) and the OR of Nipping was 6.65e-06 (95% C.I. 6.33e-10–.0698, *P* = 0.012). Moreover, the area under the ROC curve for the binary logistic regression with retinal characteristics was 0.8582, and the fitting degree of regression models was 60.48% (Prob > chi-square = 0.6048).

**Conclusion:**

This study demonstrated that retinal image analysis can provide useful information for POI identification and certain characteristics may help with early clinical diagnosis of POI.

## Introduction

Premature ovarian insufficiency (POI) is a clinical syndrome characterized by premature decline of ovarian function, abnormal menstruation (frequent or infrequent menstrual cycles, hypomenorrhea or amenorrhea) [1], increased serum gonadotropin levels and decreased estrogen levels, resulting in the decrease of reproductive function [2]. Currently, the diagnosis of POI is mainly based on clinical symptoms and serum measurements. If a woman under the age of 40 experiences the above symptoms for three consecutive months and has a persistently elevated FSH level above 25mIU/mL, the preliminary diagnosis of POI can be made. However, two or more blood tests with an interval of 4–6 weeks are required for confirmation, which can be relatively expensive [3, 4]. In addition, there are other diagnostic methods such as ultrasound examination, ovarian stimulation test, genetic testing, etc., that can be used as adjuncts to diagnosis [2]. The prevalence rate of POI in the general population was about 1% [5], and it is not rare among women of childbearing age. A recent national registry study involving 103,6918 women from Sweden showed that the overall prevalence of POI was 1.9% [6].

POI not only affects menstruation and fertility, but also leads to early menopausal symptoms and increased risk of long-term cardiovascular disease [7]. A study [8] in Korea demonstrated that the menstrual cycles of patients with premature ovarian failure (POF) may be affected by the frequencies of vascular endothelial growth factor (VEGF). It was reported that decrease in level of endogenous estrogen can lead to vascular aging, including endothelial cell damage, intimal smooth muscle cell proliferation and other symptoms affecting atherosclerosis formation [9, 10]. Therefore, POI may associate with some vessel’s diseases or lead to vascular changes.

The clinical diagnostic of POI is mainly based on clinical symptoms and serum measurements [2]. However, drawing blood for serological examination is invasive. Besides, two or more blood tests with an interval of 4–6 weeks is needed for diagnosis, resulting in a relatively high cost.

Retinal images can reflect the state of micro vessels and provide a direct observation of the changes in fundus vessels. Previous studies found a correlation between changes in fundus vessels and cardiovascular diseases [11–13]. The changes of vessels in POI patients maybe reflect in their retinal characteristics. The retinal image analysis could provide some helpful information for diagnosis of POI by monitoring how healthy their blood vessels are.

## Material and methods

### Ethical statement

This study was approved by the ethics committee of the Affiliated Shenzhen Maternity & Child Healthcare Hospital (Approval Number: SFYLS2020-012) and was performed in accordance with the guidelines of the Declaration of Helsinki (1964). This study was registered at Chinese Clinical Trial Registry (Registration No. ChiCTR2000029576).

### Data collection

**Fig. 1:**
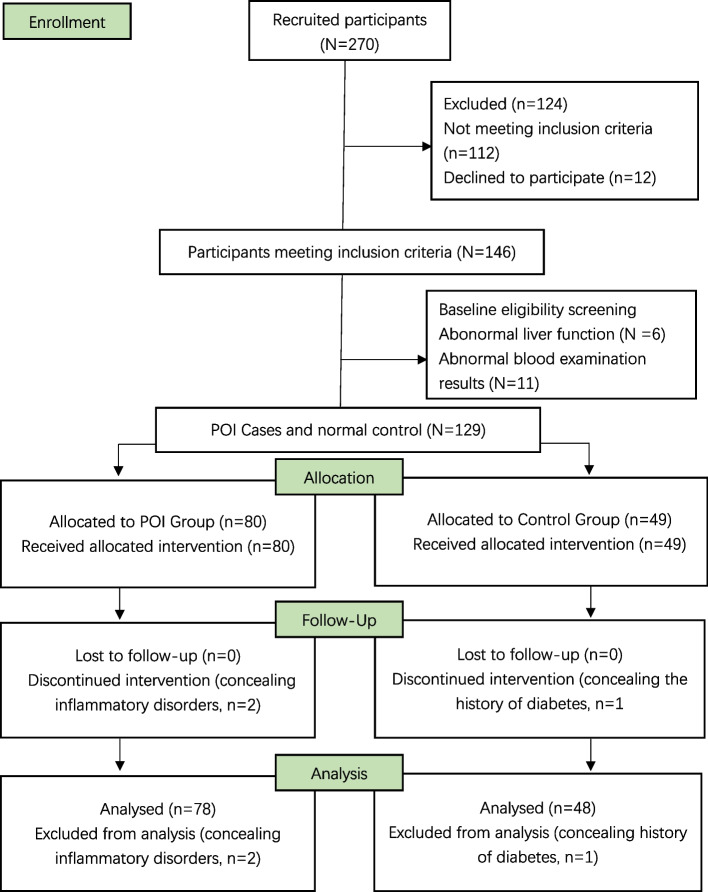
Flowchart of participant recruitment. POI represents women with premature ovarian insufficiency

In this case–control study, 78 women with spontaneous POI and 48 normal women were recruited from the Affiliated Shenzhen Maternity & Child Healthcare Hospital from August 1^st^, 2018 to October 30^th^, 2020. The average period of amenorrhea was 1.8 years (from 5 months to 4 years) in women with POI. Spontaneous POI was diagnosed according to the criteria established in the management guidelines of the European Society of Human Reproduction and Embryology: primary or secondary amenorrhea lasting for at least 4 months before 40 years old, with at least two measurements of serum follicle-stimulating hormone levels exceeding 25 IU/L with an interval of 4–6 weeks [4, 5]. Patients with karyotype abnormalities, genetic defects (such as Turner syndrome), hypothalamic amenorrhea, POI with autoimmune symptoms, infectious or iatrogenic etiologies or abnormal liver function were excluded. In order to exclude the cardiovascular disease which may influence the retinal vessel, patients with diabetes, hypertension, dyslipidemia, inflammatory disorders were also excluded in this study. 48 normal group with regular menstrual cycles and without POI diagnosis history and cardiovascular diseases was selected (Fig. 1). All participants provided written consent for their participation in the study. The age, height, weight, infertility duration of both the POI group and control group were recorded. The body mass index (BMI) was calculated. The level of follicle-stimulating hormone (FSH), level of luteinizing hormone (LH) and level of estradiol (E2) and level of anti-Mullerian hormone (AMH) of all participants were measured. The FSH/LH ratio was calculated (Table 1).

**Table 1:** Characterization of POI group and control group

Characteristics	POI Group (*n* = 78)	Control Group (*n* = 48)	*P-value*
**Age (year)**	34.397 ± 4.04	34.75 ± 4.17	0.640
**Height (cm)**	160.769 ± 4.43	160.708 ± 5.11	0.944
**Weight (kg)**	56.583 ± 4.89	56.563 ± 4.839	0.994
**Body mass index (kg/m**2**)**	21.864 ± 1.26	21.880 ± 1.126	0.943
**Infertility duration** (**year**)	2.20 ± 1.43	0.25 ± 0.67	0.000
**FSH (IU/L)**	29.362 ± 7.758	7.590 ± 5.707	0.000
**LH (IU/L)**	12.491 ± 3.051	6.027 ± 1.735	0.000
**FSH/LH ratio**	2.345 ± 0.376	1.168 ± 0.351	0.000
**E2 (pg/ml)**	31.846 ± 10.781	43.625 ± 6.111	0.000
**AMH (ng/mL)**	0.5687 ± 0.400	2.237 ± 0.756	0.000

*POI* Premature ovarian insufficiency, *BMI* Body mass index, *FSH* Follicle-stimulating hormone, *LH* Luteinizing hormone, *E2* oestradiol, *P* Progesterone, *T* Testosterone, *PRL* Prolactin, *AMH* Anti-Müllerian hormone

### Sample size

The diagnostic value of ARIA was evaluated by selecting POI patients and normal women. ARIA is expected to diagnose POI with α = 0.025, power 1-β = 0.9, AUC0 = 0.5, AUC1 = 0.7.Patients and normal women were estimated with a 1:1 sample size. By using the PASS 15 software, 41 patients and 41 normal women were required to be included, with a total of 82 subjects. To estimate a 10% loss to follow-up,46 patients and 46 normal women would need to be enrolled. But in our study, 78 POI patients meeting the inclusion criteria were all included.

### Retinal characteristics

**Fig. 2:**
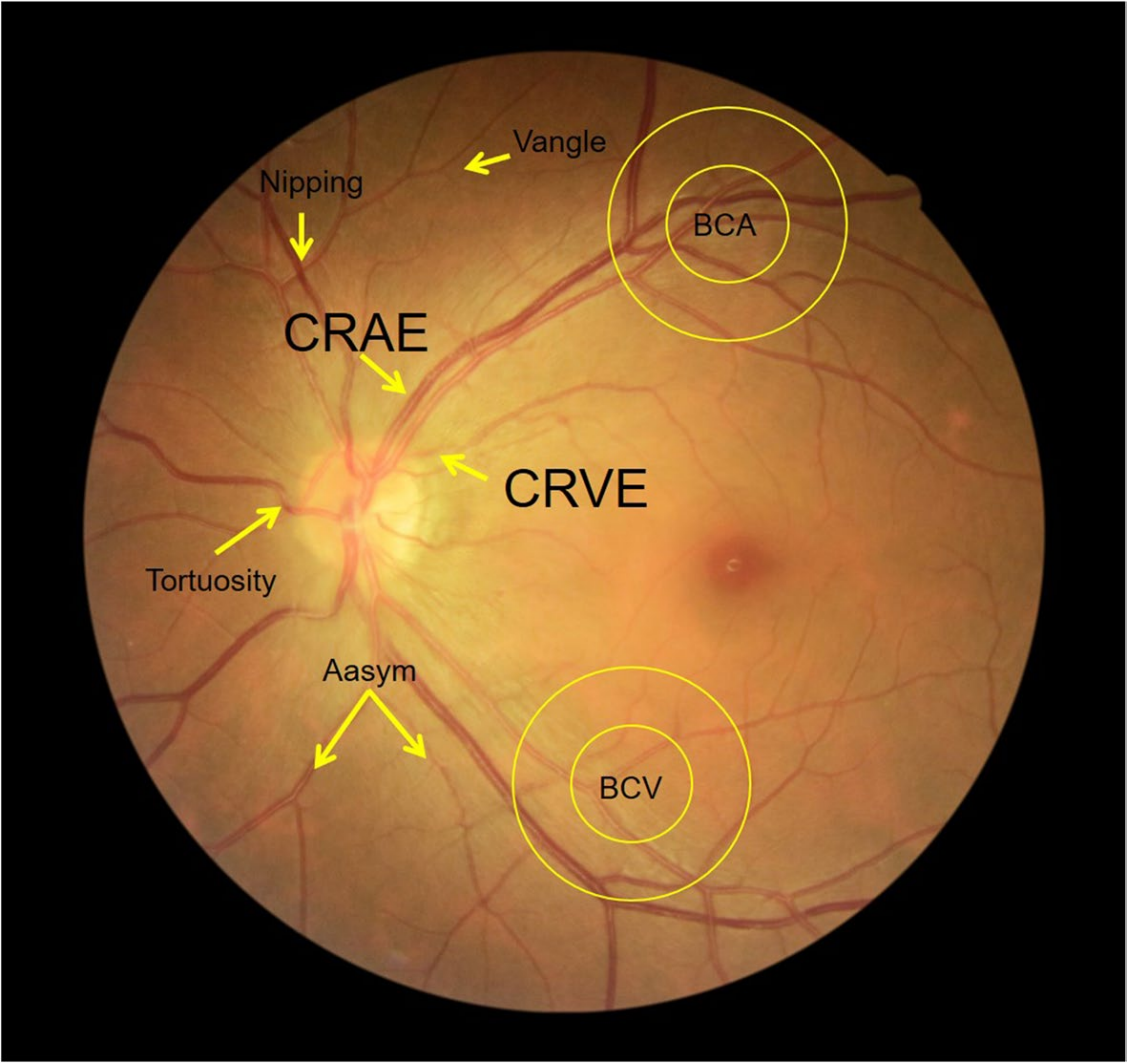
Characterization of retinal vessel in the POI and control groups.Nipping,arteriole-venous nipping;Aocclusion,arteriole occlusion;Tortuosity,tortuosity; Hemorrhage,hemorrhages; Exudates, exudates;Aasym, asymmetry index of arterioles; Vasym, asymmetry index of venules;BCV,bifurcation coefficient of venule; BCA,bifurcation coefficient of arterioles; Vangle, bifurcation angles of venule; Aangle,bifurcation angles of arterioles;CRAE,central retinal artery equivalent; CRVE,central retinal vein equivalent. AVR, CRAE to CRVE ratio; FDv,the fractal dimension of the retinal vasculature;arterioles FDa, the fractal dimension of the retinal arterioles

Retinal images were taken by a digital retinal camera (CR-2 AF, Canon, Japan). To ensure the compatibility of image parameters, retinal images were scaled to 1365 ∗ 1024 pixels and saved in jpg format. A fully automatic retinal image analysis (ARIA) system [14] using R and MATLAB was used to measure retinal characteristics. Retinal characteristics of retinal vessel in this study are arteriole-venous nipping (Nipping), arteriole occlusion (Aocclusion), tortuosity (Tortuosity), hemorrhages (Hemorrhage), exudates (Exudates), asymmetry index of arterioles (Aasym), bifurcation coefficient of venule (BCV), bifurcation coefficient of arterioles (BCA), and bifurcation angles of venule (Vangle). According to a method developed by Knudtson et al., the retinal vessel measurements were summarized as central retinal artery equivalent (CRAE) and central retinal vein equivalent (CRVE), representing the diameters of arterioles and venules, respectively [15]. The arteriole-to-venule ratio (AVR) was calculated as the ratio of CRAE to CRVE. Vessel tortuosity provides both qualitative and quantitative information by visual grading of one fovea-center and one disc-centered fundus image. Arteriole venous nipping causes narrowing of the venule when crossed by an arteriole. The arteriole occlusions refer to the blockage of blood flow inside the arterioles when obstructed by an emboli. The branching pattern of retinal vessels, such as BCV, the ratio of the widths of branching vessels to trunk vessels, the angle between two branching vessels and index of venules asymmetry (Vasym), the ratio of diameters of two branching vessels represents the relationship between the trunk and the branching vessels. The fractal dimension of the retinal vasculature (FDv) and arterioles (FDa) measured the complexity of branching patterns. The bifurcation angles of arterioles (Aangle) represent the mean of three sets of vessels in one retinal image. The analysis was performed using three sets of vessels in a single retinal image. Hemorrhage and Exudates were recorded as present or absent and their probabilities (0 to 1) were calculated (Fig. 2).

### Statistical analysis

Chi-square tests, and two-tailed independent samples t-tests were used to compare the demographics and retinal characteristics between the POI group and the control group. Retinal characteristics of patients with POI were identified using a univariate analysis. A *p*-value < 0.05 was considered as statistically significant. The binary logistic regression with backward stepwise selection was used to select the best model. The classification accuracy and the area under the curve of the receiver operating characteristic (ROC) were measured. All the data were analyzed using the Statistical Package for Social Science software Stata 15.0 (Stata Corp., College Station, TX, USA).

## Results

The general characteristics of the study participants were summarized in Table 1. The average age was 34.53(SD: ± 4.11) years, the average BMI was 21.8(SD: ± 1.13). In the univariate analysis, clinical characteristics such as age, height, weight, BMI showed no statistical significance between the two groups(*P >* 0.05). Compared to the control group, the POI group had significantly higher average infertility duration (2.20 ± 1.43 V.S. 0.25 ± 0.67; *P <* 0.001), FSH(29.36 ± 7.76 V.S. 7.59 ± 5.71; *P <* 0.001), LH(12.49 ± 3.05 V.S. 6.03 ± 1.74; *P <* 0.001) and FSH/LH ratio (2.35 ± 0.38 V.S. 1.17 ± 0.35; *P <* 0.001). There were significantly lower levels of average E2 in the POI group (31.85 ± 10.78 V.S. 43.63 ± 6.11; *P <* 0.001) and AMH (0.57 ± 0.40 V.S. 2.24 ± 0.76; *P <* 0.001) than those in the control group.

**Fig. 3:**
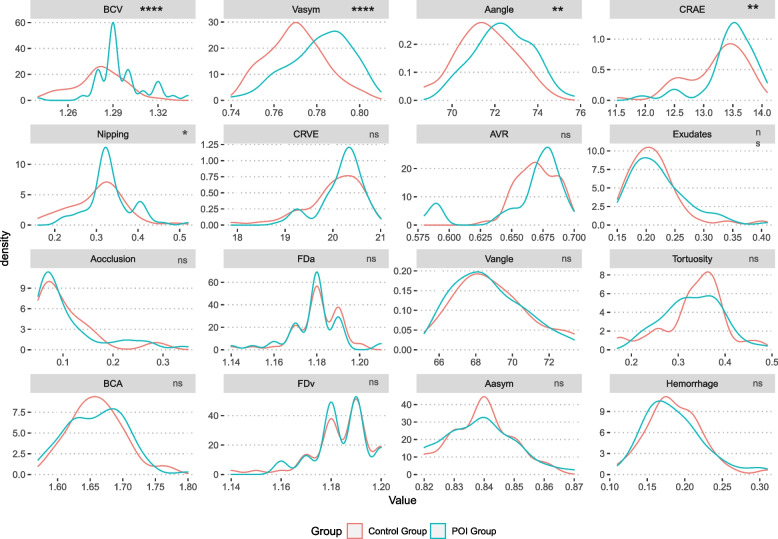
The retinal characteristics of the POI group and control group. Note: The *p*-value with a * represents nonparametric test of matched pair data (Wilcoxon Signed-Rank Test), others use paired t-test. **** represents *P* < 0.000, ** represents *P* < 0.01, * represents *P* < 0.05. The retinal characteristics that differed significantly between the POI and control groups. POI represents women with premature ovarian insufficiency

Compared to the retinal characteristics, the POI group had higher CRAE (13.46 ± 0.44 V.S. 13.22 ± 0.50; P = 0.006), CRVE (20.19 ± 0.44 V.S. 20.01 ± 0.61 P = 0.001); larger Aangle (72.37 ± 1.32 V.S.71.56 ± 1.34, P = 0.001) and BCV (1.29 ± 0.02 V.S.1.28 ± 0.02, P = 0.001); more obvious Nipping (0.33 ± 0.05 V.S.0.30 ± 0.06, P = 0.005), and lower AVR (0.66 ± 0.03 V.S 0.67 ± 0.02, P = 0.012) (Fig. 3).

**Fig. 4:**
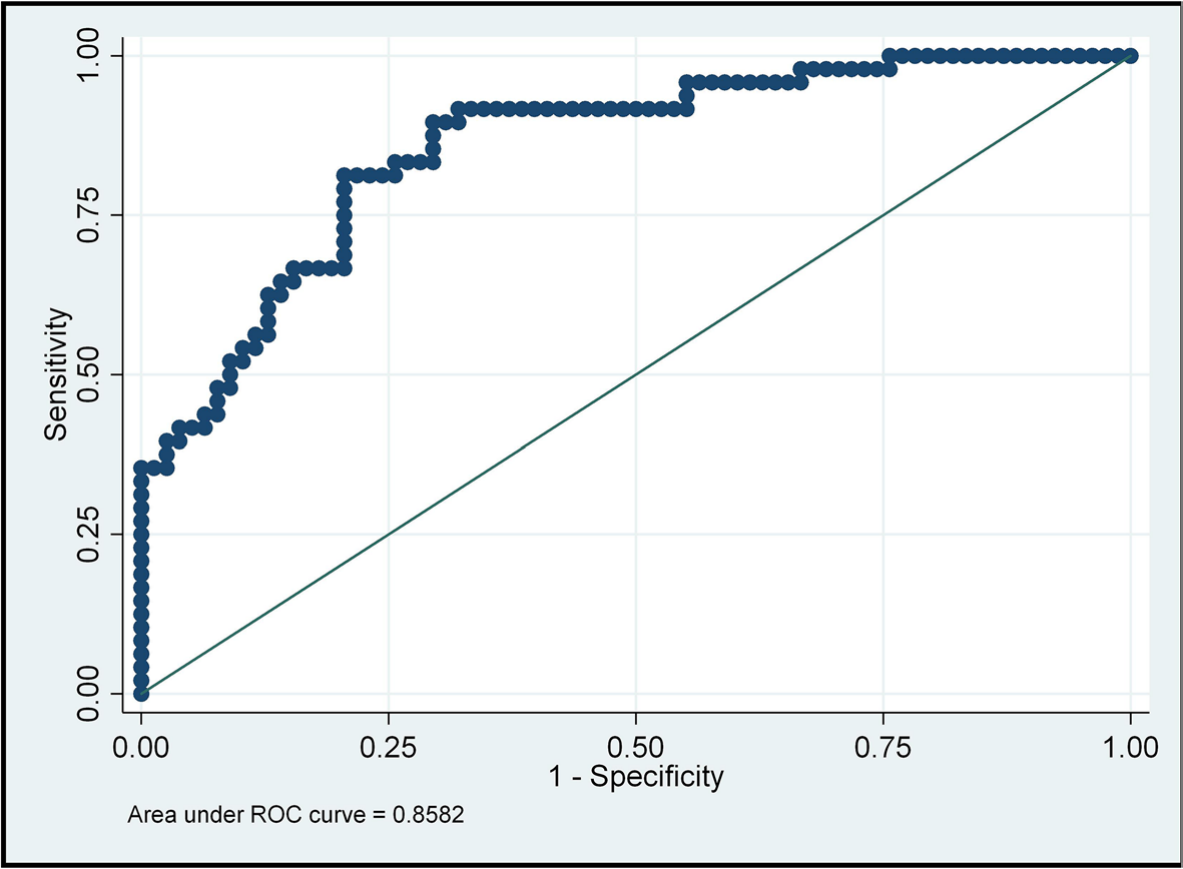
ROC curve for model classification. Note: Area under ROC curve = 0.8582, Prob > chi2 = 0.6048. The area under the ROC curve for the binary logistic regression with retinal characteristics, and the fitting degree of regression models was 60.48% (Prob > chi-square = 0.6048)

The multivariate logistic regression model was built for POI identification. In the POI group, the OR of Vasym was 6.72e-32 (95% C.I. 4.62e-49–9.79e-15, *P* = 0.000), the OR of BCV was 5.66e-20 (95% C.I. 1.93e-34–0.0000, *P* = 5.66e-20) and the OR of Nipping was 6.65e-06 (95% C.I. 6.33e-10–0.0698, *P* = 0.012) (Table 2). Diagnostic testing was performed to evaluate the prediction accuracy of the model. The area under the ROC curve for the binary logistic regression with retinal characteristics was 0.8582, and the goodness of fit test was 60.48% (Prob = 0.6048, Prob > chi-square) (Fig. 4).

**Table 2:** Results of the binary logistic regression

**Group**	**Odds Ratio**	Std. Err	z	***P-value***	**[95% Conf. Interval]**	
**CRAE**	0 .3892482	0.2159073	-1.70	0.089	1.312454	1.154434
**Vasym**	6.72e-32	1.36e-30	-3.56	0.000*	4.62e-49	9.79e-15
**Aangle**	0.7426158	0.1383556	-1.60	0.110	0.110	0.5154401
**BCV**	5.66e-20	9.61e-19	-2.61	0.009*	1.93e-34	0.000166
**Nipping**	6.65e-06	0 .000314	-2.52	0.012*	6.33e-10	0.697619
**_cons**	1.37e + 65	4.00e + 66	5.14	0.000*	2.03e + 40	9.23e + 89

The *p*-value with a * represents nonparametric test of matched pair data(Wilcoxon Signed-Rank Test),others use paired t-test

*Nipping* Arteriole-venous nipping, *Aocclusion* Arteriole occlusion, *Tortuosity* Tortuosity, *Aasym* Asymmetry index of arterioles, *Vasym* Asymmetry index of venules, *BCV* Bifurcation coefficient of venule, *BCA* Bifurcation coefficient of arterioles, *Vangle* Bifurcation angles of venule, *Aangle* Bifurcation angles of arterioles, *CRAE* Central retinal artery equivalent, *CRVE* Central retinal vein equivalent, *AVR* CRAE to CRVE ratio, *FDv* The fractal dimension of the retinal vasculature, *arterioles FDa* The fractal dimension of the retinal arterioles

## Discussion

Luteinizing hormone, follicle-stimulating hormone and estradiol are the common markers in POI patients. POI indicates an early menopause and it can be diagnosed when the levels of blood LH and FSH increase substantially. One of the most sensitive hormones to diagnosis POI is FSH, which negatively correlated with ovarian function. Therefore, monitoring of elevated FSH for several months is helpful for women to be diagnosed of early-onset ovarian insufficiency. The ovaries occasionally experience a stage of low activity, and then return to normal function. For this reason, to diagnosis POI, at least two hormone measurements from two menstrual cycles are required. Furthermore, time limit to blood test is important, considering that the FSH and LH levels in women vary greatly in each menstrual cycle, requiring blood samples to be drawn within 3–5 days after menstruation. In a word, the diagnosis using blood examinations is invasive and inconvenient. It is very important to propose an effective, noninvasive diagnostic method for patients with POI in clinical application.

POI not only causes menstrual changes, but also affects blood vessels. E2 has a direct stimulatory effect on vascular endothelial cells via the estrogen receptor [16, 17]. It was reported that the endocrine changes in female patients, especially the decrease of E2, were associated some abnormalities of the microvascular system [18]. Retina is a small part of the vascular system in human body which can be observed directly. It is not only an important sign of retinopathy, but also a direct evidence of vessels injury in human body. Some endocrine diseases, induced by hormone changes, can affect the vascular system, and can be reflected in the fundus blood vessels. Raizada [19] found that the change in sex hormones can cause the retinal capillaries, arterioles and venues to develop mild per phlebitis. E2 have been linked with alterations in the ocular and retinal circulation, Estrogen-replacement therapy in postmenopausal women can substantially reduce vascular resistance distal of the ophthalmic artery to levels matching those of young women [20]. LH plays a physiologic role in VEGF regulation and retinal vascularization in the development of eyes but it is not required for the development of various components of retina in mouse [21]. In the vasculature of nonmalignant tissues, FSHR is only expressed in placental endothelial cells [22] and to a lesser extent, in the endothelium of ovaries [23] and testis [24]. Estrogen has dual effects on the inflammatory system, with anti-inflammatory and pro-inflammatory effects, which increase after menopause [25, 26]. Inflammatory aging plays an important role in the pathogenesis of POI, leading to the imbalance of inflammatory cytokine network [27], so it can be inferred that POI has a close relationship with vascular endothelial dysfunction and vascular inflammation due to low estrogen level, and this result can be confirmed that there is no sign of accelerated vascular aging in POI women receiving timely and sufficient HRT. Therefore, endothelial function is a barometer of vascular health and a predictor of atherosclerosis, which may begin earlier than usual in female patients with POI [28, 29]. Kalantaridou et al. [30] found that after receiving HRT 6 months, the blood flow mediated diastolic function in POI patients increased more than twice and returned to the normal value similar to that in the control group. It can be inferred that the vascular endothelial function can be effectively reflected by the vascular diastolic function. In our study, we found that POI is related to the increase of risk factors or markers reflecting vasodilation function in retinal fundus vascular analysis, such as Vasym, BCV and Nipping, which is consistent with the author's purpose to distinguish POI from healthy people by observing retinal fundus vessels.

Ocular blood flow velocity was positively correlated with levels of LH, FSH and total cholesterol. Therefore, the characteristics of retinal image can provide useful information for the diagnosis of POI. In our study, we found that the characteristics of retinal image of POI were mainly focus on the vein vessels. The increased branching coefficients of arteries and venules were associated with a higher risk of POI, indicating changes in retinal vascular branching patterns. In healthy individuals, retinal vessels provide optimal pathways for blood flow, and thus the energy consumption is minimal. Therefore, we hypothesized that the retinal microcirculation abnormalities in POI patients were associated with chronic and lower levels of E2.

So far, there was no literature supporting the finding that the protective effect of estrogen on cardiovascular system is mainly through the influence of coagulation and fibrinolysis system, inflammatory factors and vascular endothelial cells [19, 31–33], which may reflect that microvascular injury is caused by POI and can be identified by retinal bifurcation coefficient. In previous studies, it was denoted that early menopause age is associated with higher CVD risk and POI patients have higher CVD risk and CVD mortality compared to the general population [34–36]. Detection of subclinical atherosclerosis may be an important approach in the prevention of cardiovascular events in these young women with POI. Recent studies have shown that the pathogenesis of early-onset atherosclerosis in ovarian lesions (ovarian aging related diseases, such as POI) is related to ROS induced DNA changes, mitochondrial dysfunction and reduced ATP production. Oxidative stress is the focus of this study, which also suggests the correlation between women with POI and subclinical atherosclerosis. Fundus vessels can reflect systemic vascular conditions, including subclinical arteriosclerosis [37]. The study found POF patients exhibited significantly different frequencies of the VEGF genotype [8], it may influence the level and types of VEGF to affect angiogenesis and angiogenesis of blood vessels. Since biological effects are considered to be more complex, we explored the interaction between retinal features. This study found that the risk of POI increased as the levels of a series of retinal components (CRAE, CRVE, BCA, venous angle, arterial angle, FDA) increased.

## Limitation

This study has some limitations. All participants in the study were recruited from the same hospital, which may lead to potential selection bias. Therefore, multicenter clinical studies are necessary in further research. In addition, cross-sectional design resulted in difficulties in causal inference. A larger sample size is needed in further research.

## Conclusion

The retinal imaging can provide valuable diagnostic information for patients with POI. Our study has shown that women with POI have special retinal features such as wider retinal arterioles and venules, and had larger bifurcation angles of arterioles These changes are thought to be related to alterations in the hormonal environment that occur with POI. However, the changes observed in the retinal vessels and other retinal structures can be non-specific, it may be present in other conditions, such as hypertension and diabetes. The aims of our study were to demonstrate that retinal image analysis can provide useful information for POI identification and certain characteristics may help with early clinical diagnosis of POI. It is important to comprehensive consider other clinical factors, such as age of onset of menopause symptoms, family history, and laboratory testing, to arrive at a definitive diagnosis of POI.

## Supplementary materials

These are the retinal images of 126 participants recruited in the study. Supplementary for univariate analysis.

## Data Availability

The EXCEL data used to support the findings of this study are available from the corresponding author upon request.
